# Exercise-induced myokine FNDC5/irisin functions in cardiovascular protection and intracerebral retrieval of synaptic plasticity

**DOI:** 10.1186/s13578-019-0294-y

**Published:** 2019-04-03

**Authors:** Xin Zhou, MengMeng Xu, Joseph L. Bryant, Jianjie Ma, Xuehong Xu

**Affiliations:** 1 Laboratory of Cell Biology, Genetics and Developmental Biology, Shaanxi Normal University College of Life Sciences, Xi’an, 710062 China; 2 Ohio State University College of Medicine, Columbus, OH 43210 USA; 3 Medical-Scientist Training Program and Department of Pharmacology, Duke University Medical Center, Durham, NC 27710 USA; 4 University of Maryland School of Medicine, Baltimore, MD 21287 USA

## Abstract

Physical exercise is well known to benefit human health at every age. However, the exact mechanism through which physical exercise improves health remains unknown. Recent studies into exercise-induced myokine FNDC5/irisin, a newly discovered hormone, have begun to shed light on this mystery. Exercise-induced myokine FNDC5/irisin have been shown to be protective against cardiovascular damage post ischemic event, improve function in the neurons of Alzheimer’s disease patients, and have been implicated in macrophage and adipocyte regulation. Elegantly designed experiments have shown FNDC5/irisin to promote Nkx2.5^+^ cardiac progenitor cell dependent cardiac regeneration, neovascularization, and reduce cardiac fibrosis. It has also been shown to improve macrophage function, which may protect against injuries to the cardiac conduction system. Similarly, FNDC5/irisin knockout mice have been shown to have reduced memory performance, while peripheral overexpression of FNDC5/irisin has been shown to improve memory impairment in a murine Alzheimer’s disease model. Finally, FNDC5/irisin has been linked to regulation of osteocytes and adipocytes by signaling through the cytoplasmic membrane integrated protein aV/b5 integrin, the first known receptor for this newly discovered hormone. Although these recent discoveries have cemented the importance of FNDC5/irisin, many details regarding how FNDC5/irisin fits into the physiology of exercise benefits remain unknown and are deserving of future inquiry.

Routine physical exercise is well known to improve human health at every age. However, the molecular mechanism behind the benefits of exercise remains largely unclear. In recent years, a newly discovered hormone, FNDC5/irisin, became a promising target for the link between physical exercise and health [1, 2]. FNDC5/irisin is produced when exercise promotes splicing or cleaving of the myocyte extracellular domain fibronectin type III domain-containing protein 5 (FNDC5), a membrane integrated precursor protein controlled by myocyte peroxisome proliferator-activated receptor-γ coactivator 1α (PGC-1α). Elevated levels of this exercise induced FNDC5/irisin has been linked to cardiovascular protection/repair and improved function in neurons of Alzheimer’s disease, with new findings hinting at involvement in osteocyte and adipocyte regulation.

Two independent clinical studies have cemented the protective effects of physical activity against cardiovascular disease (CVD) [3, 4]. In the PURE study, the incidences of and morality from myocardial infarction, stroke, and heart failure in a 130,000 cohort from 17 high-income, middle-income, and low-income countries was significantly lower in the group that engaged in regular physical activity. Interestingly, this study found no difference between recreational and non-recreational physical activity on the protective effect against CVD disease and mortality [3]. Similarly, a second study on 487,000 Chinese men and women found physical activity to be equality effective between the sexes at preventing vascular events, including CVD, ischemic stroke, intracerebral hemorrhage, and deaths from vascular events [4]. These findings have been further confirmed in multiple animal models. A mechanistic investigation has found FNDC5/irisin to promote cardiac regeneration, neovascularization, and reduce cardiac fibrosis by amplifying the ability of Nkx2.5^+^ cardiac progenitor cells (CPC) to repair cardiomyocytes and promote proliferation after an ischemic event [5]. This protective effect has been shown to mediated by FNDC5/irisin induced up-regulation of proliferative markers Ki67 and phosphorylated histone 3 and marked reduction of histone deacetylase 4 and increased p38 acetylation in treated CPCs [6]. FNDC5/irisin may also amplify healing by improving macrophage function through FNDC5/irisin triggered oxidization of low density lipoproteins via the endoplasmic reticulum stress pathway. This, in turn, maintains the cardiac conduction system as macrophage are known to facilitate electrical conduction and syncytium calcium signaling in the heart [6–8].

Analysis of Alzheimer disease (AD) patients and accompanying studies utilizing AD animal models also linked exercise-induced FNDC5/irisin to improved synaptic plasticity in symptomatic patients and animals [9]. De Freitas and collogues first found FNDC5/irisin to be expressed in the hippocampus, cortex, and cerebrospinal fluid of wild-type C57BL/6 mice with lower levels found in AD mice. Wild-type mice with FNDC5/irisin knocked out in the brain using FNDC5/irisin-specific shRNA constructs showed impaired long-term potentiation, which led to poorer performance in novel object recognition, radial arm water maze, and contextual fear conditioning. Conversely, an intracerebroventricular infusion of FNDC5/irisin rescued memory impairment in AD mice. These findings were further proven by the ability to rescue memory impairment through peripheral overexpression of FNDC5/irisin and the ability to reduce the neuroprotectivity of physical exercise through the blockade of peripheral or brain FNDC5/irisin [9]. This seminal paper was the first to demonstrate a mechanism behind the protective capacity of physical exercise in AD patients, which will indubitably inform future clinical approaches to maintain and rescue synaptic plasticity in AD patients.

Recent work has also found FNDC5/irisin to be important in the regulation of osteocytes and adipocytes. Using a FNDC5/irisin precursor knockout mouse model, Spiegelman and colleagues proved that genetic deletion of FNDC5/irisin completely blocks osteocytic osteolysis. FNDC5/irisin involvement in this signaling was shown to be through binding to αV/β5 integrin, a bone remodeling regulation receptor found on the surface of osteocytes [9, 10]. They similarity showed FNDC5/irisin to be important to the white to brown conversion of adipocytes [9] (Fig. 1, left). However, the exact mechanisms through which FNDC5/irisin improves cardiac, central nervous system, and other organs function remains largely unknown (Fig. 1, right). As previously discussed, circulating macrophages are affected by FNDC5/irisin and may account for some of the positive effects exercise plays on most organ systems. However, the effect of FNDC5/irisin on the brain is especially unknown as the brain is an immunologically privileged space protected by the blood–brain barrier (BBB). Thus, FNDC5/irisin likely crosses the BBB to play a role directly in the central nervous system. Although recent discoveries have cemented the importance of FNDC5/irisin in exercise-induced health benefits, there remains many unexplored facets regarding its exact mechanisms of action.

**Fig. 1:**
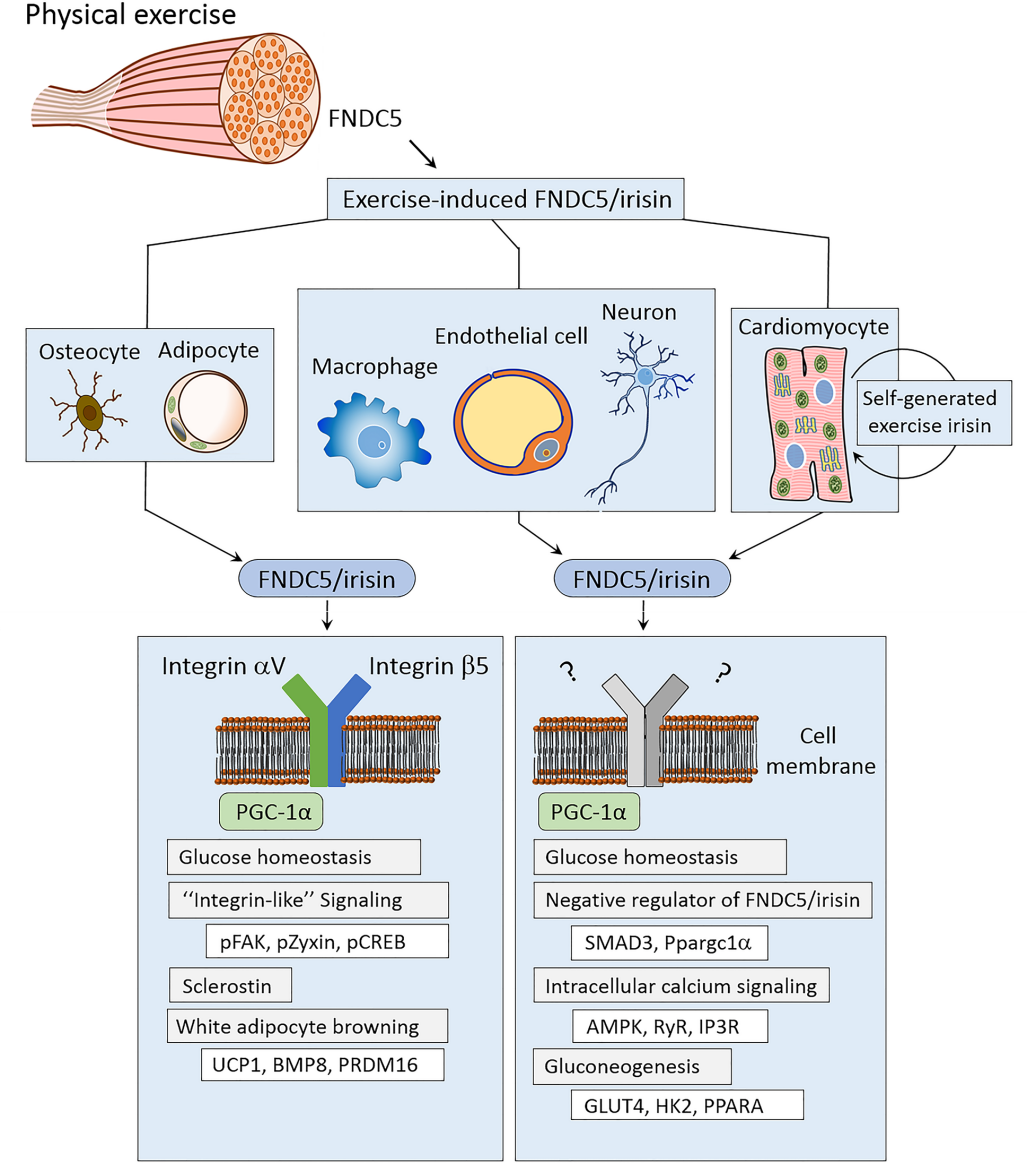
Exercise promoted FNDC5/irisin formation and signaling. FNDC5/irisin has been shown to affect signaling of osteocytes and adipocytes through the pathways shown on the left and macrophages, endothelial cells, neurons, and cardiomyocytes through the pathways shown on the right
